# Pearls of Wisdom from Teaching Rounds: Reconceptualizing “See One, Do One” as Social Learning

**DOI:** 10.1007/s40670-024-02184-9

**Published:** 2024-10-01

**Authors:** Jessica R. Newman, Dorothy Hughes

**Affiliations:** 1 Division of Infectious Diseases, Department of Internal Medicine, University of Kansas Medical Center, 3901 Rainbow Blvd. Mailstop 1028, Kansas City, KS 66160 USA; 2 Department of Population Health, University of Kansas School of Medicine, Salina, KS USA

**Keywords:** Teaching rounds, Social learning, Faculty traits

## Abstract

**Background:**

Experiential education is paramount to clinical instruction in medicine. During teaching rounds, faculty can assess medical knowledge and clinical reasoning while modeling evidence-based patient care and effective communication. This study aimed to describe observed traits common across faculty who contribute to favorable learning environments.

**Design:**

Students completed an Internal Medicine (IM) Student Chief elective at a midwestern United States Academic Tertiary Care Center in which M4s aided Clerkship leadership in near-peer teaching and communicating with students. Chiefs participated in rounds for experiential learning in clinical teaching. For this qualitative, descriptive study, M4s were asked to provide at least three “pearls of wisdom,” on observations about teaching and learning in reflective essays during two consecutive academic years. Twenty-nine M4s participated in the elective and submitted 238 pearls. The research team undertook document review and qualitatively analyzed content of Chiefs’ reflective essays. Detailed codes were organized into primary and secondary themes by each author, and then re-evaluated until they reached consensus. Through thematic analysis, we identified faculty traits students found conducive to learning.

**Results:**

Three primary themes emerged: Attributes, Autonomy, and Achieving Engagement.

**Conclusion:**

Experiential educational encounters are transformative to developing new physicians. Chiefs highlighted positive aspects of teaching rounds described previously including providing clear expectations, promoting goal setting, engagement, and instilling confidence. They also valued humor, recognizing cognitive overload, displaying humility, listening skills, and mentorship. While one attending cannot replicate all described effective techniques simultaneously, faculty development that promotes refining these approaches may improve the clinical learning environment for all learners.

## Introduction

Since the practice of medicine began, training new physicians has relied on experiential education. This apprenticeship style of skill acquisition describes a person learning a trade or acquiring abilities from an experienced person. The term apprentice itself was derived from the Medieval Latin “apprehend,” meaning, “to learn,” and the Old French “aprentiz” translated to “someone learning.” This model of instruction in medicine has matured over time, but Halsted’s model of “see one, do one, teach one” still describes the increasing autonomy a physician-in-training takes on in today’s medical school and residency educational programs [1].

As Dr. Salib eloquently stated, “I envision teaching rounds as a finely choreographed dance—everything must work together seamlessly for an effective and well-balanced end product.” [3]. This “product” must include providing high-quality patient care at its most basic level, but also providing expectations to team members, promoting goal-setting and achievement, engagement, and enthusiastic discussion among all team members, promoting intellectual curiosity and confidence, and doing all this efficiently and effectively [3]. Medical clerkships (required M3 core rotations) and M4 student rotations, such as sub-internships, are often based around patient care and teaching rounds, with supplemental self-directed modules, simulation experiences, and formal (lecture) and informal (chalk talk) didactics. Teaching related to patient care can be done away from the patient or at bedside (done in the presence of the patient). Although ward rounds or teaching rounds can make up as much as 31% of resident teaching, bedside teaching has decreased in the past few decades, making up 17–19% of clinical medical training [4–6]. Experienced academic hospitalists argue this bedside time models professionalism and humanist behaviors as well as promotes clinical reasoning skills and teamwork [7, 8]. On teaching rounds, faculty can better assess learner medical knowledge and clinical reasoning skills, assess autonomy and communication skills, and establish a team culture that can promote continuation of care in the attending’s absence, as the attending may only be physically present with the team 15 h per week [8, 9]. The purpose of rounds has been investigated in semi-structured focus groups with attendings and medical students and via surveys; this previous research often highlights that medical education, patient care, and assessment and evaluation are important components [10, 11].

Evaluating clinical educators on specific teaching behaviors can be challenging. A 2002 survey of 2250 M4 students found that seeing new patients with teaching teams, spontaneous talks, prepared teaching presentations, and engagement in discussions in clinical care were positively associated with satisfaction ratings for attending physicians whereas seeming “rushed” was negatively associated with satisfaction [12]. Answering specific patient care questions, teaching on topics chosen by learners, and feedback were also found to be performed more often by attendings when teaching rounds was considered effective. Other positive descriptors for rounds include enthusiasm, encouragement, inspiring confidence, communication skills, clinical competence, and creating a safe learning environment [13]. Measuring these favorable traits and actions is difficult. A 2010 study attempted utilization of a 15-point checklist to create a composite mean score of observable clinical teaching behaviors. While generalizability of ratings was acceptable, inter-rater reliability varied between occasions and items [14]. This illustrated difficulties in evaluating excellence in clinical teaching. Without effective evaluation, reproducibility is a challenge limiting ability to distill best practices [15–19]. There is also disagreement between learners and peer faculty members on what attributes are most important in clinical teachers, though a Clinical Teaching Perception Inventory has attempted to align key characteristics among resident and attending teachers [15, 16, 20].

Utilizing our institution’s model of Internal Medicine teaching teams, this study aimed to identify attributes, behaviors, and techniques of teaching associated with observed positive experiences on academic hospitalist teaching rounds.

## Methods

At our institution, a 900-bed (approximately 600 acute care medical/surgical beds) midwestern Academic Tertiary Care Center, M4s may be selected to participate in IM Student Chief elective in which these M4s would aid the IM Clerkship leadership in organizing study tools and communicating with M3 learners. Primarily, they would aid in near-peer teaching and coaching for clerkship students. The IM Student Chief would also acquire experiential benefits from their teaching and coaching roles, as they had no direct patient care responsibilities during the elective. On 2 to 3 days per week, the M4 was assigned to existing inpatient teaching teams consisting of academic hospitalists, IM residents, and M3 clerkship students to observe and model more senior clinician teachers. Chief students were given an asynchronous, modular curriculum (using an online platform) on common medical education topics such as expectations, feedback, 1-min preceptor, generational learners, and chalk talks. They completed these modules during their elective but were not provided with any specific background information on attributes or behaviors associated with effective clinical teachers beforehand.

After rounding experiences, Chiefs were asked to describe at least three “pearls of wisdom” in reflective essays, sharing what they learned about teaching and learning during six to eight clinical teaching round observations. This exploratory, descriptive qualitative study utilized these “pearls of wisdom” documents as its primary data source. Participants included M4 students participating in the Student Chief Elective rotation. For their “pearls” to be included in the study, students must have completed the elective at our midwestern Academic Tertiary Care Center during the 24-month period from May 2021 to May 2023.

Following Braun and Clarke’s approach to thematic analysis, the first author reviewed data and then generated initial codes via inductive coding [21, 22]. The second author suggested additional codes, and the authors iterated the codebook. Both authors independently performed detailed coding, reviewing, and revising the codebook as appropriate. Detailed codes were organized into primary and secondary themes by each author, and then re-evaluated until they reached consensus. The primary and secondary themes are presented here with illustrative quotes (Table 1). Quotes are identified in italics with bracketed letters: [A]. Abridged data are represented by: […]. The Institutional Review Board at the University of Kansas Medical Center approved this study.

**Table 1:** Illustrative quotations from M4 student chiefs

Primary themes	Secondary themes	Illustrative quotes
Attributes	None	*“Dr. [X] is a very funny person, and I saw how this made most of the students more comfortable with presenting to [them]. But I also appreciated that [they] noticed one student not responding to this very well. [They were] able to adapt to a more serious demeanor for this student. I tend to let my humor show, and it was good for me to see how [they] managed this situation.”* [S] *“[O]ne of the attendings […] was able to create a light yet energetic atmosphere while on rounds even though we were dealing with patients who were in sad and depressing situations with unfavorable outcomes. The ability to be able to make sure your colleagues are there and present with you and not being overwhelmed by patient circumstances is a skill that may come natural to some but must be practiced by others. I felt that the students were very much enjoying rounds because of this.”* [Y]
Autonomy	None	*“Dr. [X] would have the interns present to the senior resident instead of presenting to [them]; this gives the senior resident more autonomy and helps them grow as a team leader.”* [U] *“Pushing students to re-evaluate a patient’s care plan with new data on rounds challenges students to make real time decisions in a controlled setting and to think critically about the patient’s management/diagnosis instead of falling into a potential fallacy of continuing a previously made plan.”* [I]
Achieving Engagement	Using Teaching Techniques	*“Using the Socratic method of asking ‘What do you think?’ as a response to questions allows the teacher to identify the learners understanding gap. This was best followed by an explanation or a reference to find the explanation.”* [BB] *“Dr. [X] identified one high yield learning topic to focus on for each patient each day during rounds. [Another Dr.] would give students topics to self-learn and the report back later in the day. Although not realistic on all teams/settings, I found it to be effective as it encourages self-exploration and practice with navigating resources to find medical information.”* [H]
	Modeling Behaviors	*“Dr. [X] was great at being efficient with [their] teaching and with patients. [They] balanced so well with a team capped at its patient max. I still felt like [they] built report and spend [sic] adequate time with [their] patients recognizing who may need more counseling for the day and prioritized time there. I really respected […they] always make sure the patients understood what was going on […] and that all of their questions were answered.”* [G] *“If you don’t know the answer to something it is ok to say so and better yet, you can use that moment as an opportunity to show students how you research the answers to your clinical questions.”* [Z]
	Setting Clear Expectations	*“[Attending] challenged the students to do further reading at home as well. [Their] rounds were the most formal out of all of the rounds that I attended. While this extra formality was a bit unnerving to some of the students, it also helped establish very clear expectations.”* [B] *“What I appreciated […] was the emphasis [they] placed on setting expectations. Every morning prior to the start of rounds [they] would explain how rounds would run that day (Senior taking over as the “attending” for the day, split rounds, *etc*.), who the students should direct the presentation to, what format of presentation he wanted the students to try, and what the overarching educational theme of the day would be. This only took 30 s, but went so far with ensuring everyone was on the same page for the day.”* [O]
	Creating Connections	*“The attending made a point to get to know every person’s name in the room and had us introduce ourselves to the team. This is helpful for understanding roles and everyone feeling included.”* [P] *“[Attending] would frequently stay in the resident workroom with the residents and students after rounds, if able. This allowed the learners to get to know the attending better as conversations were not only about patient care, but also made the attending accessible for questions about patient care. The learners were also given more opportunities to be a greater part of the “behind the scene” aspects of patient care like following up with consultants and problem-solving with social work. […] I noticed that rounds on the day following this was more comfortable as both the students and attending felt more comfortable with each other’s personalities and expectations.”* [H]

### Role of the Funding Source

No funding source was utilized in this study.

## Results

Twenty-nine M4 students participated in the IM Student Chief elective during academic years 2021–2022 and 2022–2023. In total, 17 (59%) men and 12 (41%) women participated. All submitted reflective essays, each of which included at least three rounding “pearls of wisdom.” The students’ pearls totaled 238. Our analysis revealed what M4s found important in how IM academic hospitalists conducted teaching rounds. The following three primary themes emerged: Attributes, Autonomy, and Achieving Engagement. First, we describe what faculty attributes students found notable in the learning environment, and second, student observations of how faculty encouraged autonomy in students and trainees. The Achieving Engagement theme included four secondary themes: using teaching techniques, modeling behaviors, setting clear expectations, and creating connections. During analysis, we noted other points that did not fit neatly into any of the three primary themes. We discuss these at the end of this section, under “Additional Insights.”

### Attributes

Students shared that attributes such as mood, demeanor, humor, empathy, and honesty can affect the learning environment and students’ ability to learn. The M4s shared that a faculty’s *moods “sets the tone for the day”* [T], and they can be contagious, lowering or uplifting clinical team members’ moods. As one M4 said, *“The leader’s mood [or] demeanor has a profound influence on the tone of rounds and the quality of learning and engagement”* [P]. Similarly, the faculty’s willingness to have fun and use humor can make them seem approachable, making learners more likely to actively participate. However, when humor is used to belittle, it has the opposite effect.

Students also reported appreciating empathy, patience, and kindness; these traits enhanced the learning environment. For example, patience allowed students time to think through answers and respond optimally. In addition, these were types of behaviors students admired and wanted to emulate. For example, *“[Attending] would offer to help the patient with anything they need in the room, like making their coffee and getting them some water”* [W].

Appreciation for honesty was often connected to other themes, such as Setting Clear Expectations under Achieving Engagement. For example:

*At one point an intern was struggling with their presentations, and [attending] was pretty blunt that [they] expected them to be performing at a higher level. [They] didn’t say it in a malicious way, just very matter of fact. This is something I especially took note of because I tend to be conflict-averse, and I thought [they] did a good job of stating […] expectations without making it personal or overly negative.* [A]

Students observed honesty being demonstrated, and they saw its utility in learning, as well is its more abstract importance to integrity.

In addition, students noted when attendings were humble and respectful. Demonstrations of humility, such as being willing to admit, “I don’t know,” led to faculty and students looking up information and learning not only clinical facts together, but also learning more about the process of researching a question.

In their “pearls of wisdom,” students focused on positive attributes that facilitated learning, but they were also able to points out negative attributes, like belittling or condescension.

### Autonomy

The M4s who participated in our study found that “good” faculty encouraged progressively greater autonomy in medical students and other trainees. They found this progressive trust prompted more critical thinking, helped instill confidence, and assisted in setting clear expectations.

Students observed attendings not only allowing them more autonomy but also allowing it in other trainees, mostly residents. For example:

*[Attending] often let the senior resident provide most of the feedback to student[s] and do most of the teaching. Furthermore, [they] had the senior also lead the conversations with the patients. I could tell that this approach helped strengthen the confidence of the resident as [they were] able to gain experience leading rounds.* [B]

Actions supporting trainee autonomy and making students comfortable giving patient presentations had the added benefit of allowing students to practice important communication skills.

### Achieving Engagement

To achieve engagement, M4s reported that faculty utilized a variety of techniques. In our analysis, we grouped these techniques into four secondary themes. We describe how faculty used effective teaching techniques, modeled behaviors for students to adopt, set clear expectations (and how students appreciated that), and created connection among clinical team members.

#### Using Teaching Techniques

Students articulated several teaching techniques they observed faculty using, including bedside teaching, active learning, closed-loop communication, and Socratic questioning. They also discussed how these techniques prompted critical thinking, created connection among the care team members, and allowed for exploration and reflection. One example of active learning that promoted critical thinking and understanding was:

*[Instructor] had […] students each prepare a handout over pulmonary hypertension and hepatorenal syndrome for everyone to have which was great because the students expressed how they felt so much more comfortable with the topics from preparing those handouts versus just hearing the information.* [K]

Another notable teaching tactic was what we termed *“key takeaways”* [C]. Students appreciated attendings boiling down complex information and conveying it verbally in short *“mini lessons”* [Q] or in writing, like a *“cheat sheet”* [S].

#### Modeling Behaviors

Through their “pearls of wisdom,” students described three behaviors they thought it was especially important to model: superior quality patient care, humility, and being evidence-based. Demonstrating humility not only promoted learning, as described under the Attitudes theme, but here we point out its role in professionalization, showing students what kind of professional they may want to be. Around evidence-based care, students found it particularly helpful for attendings to verbalize what actions were their own preference and which were grounded in evidence.

#### Setting Clear Expectations

Students’ appreciation for the setting of clear expectations had numerous benefits. They felt it helped instill confidence in learners, build autonomy, and it helped them understand how to achieve necessary objectives. One student said, *“Clear expectations make everything smoother. If the students knew how the attending approached rounds, they felt more confident in their presentations and care”* [Y].

#### Creating Connection

Students observed that attendings who strove to create connection among care team members were also approachable. These physicians were able to create effective teams. One student described how debriefs after awkward situations give *“space”* after *“awkward situations”* and help build rapport [CC]. Another student shared:

*Dr. […] would tell stories from his own training days to make connections with the residents and students and show them that mistakes are made along the way and sometimes it is necessary to learn. I think it made a more comfortable connection with the students in regard to feedback when they were wrong about plans.* [D]

It was clear that this subtheme was linked to the modeling behaviors subtheme and the Attributes primary theme. Creating connection occurs when attributes are utilized and/or behaviors are modeled.

### Additional Insights

Students also appreciated a number of faculty behaviors that did not fit well into our identified primary and secondary themes. However, we wanted to share them in the interest of facilitating greater understanding of this subject. Students appreciated when faculty recognized cognitive overload in learners and wrapped up teaching rounds, shortened chalk talks or adjusted time for teaching accordingly. They also appreciated helpful feedback, being organized, listening, mentorship, reviews of terminology, and efforts to teach to learners’ differing skill levels.

## Discussion

This was a single-center study with a small participant group. Students were asked to submit at least three pearls, though no limitation was placed on quantity. Thus, some students submitted more, and these students’ perceptions may be weighted more heavily in the analysis of themes.

Early experiential educational encounters are transformative to developing new physicians, who, even after completing training, will remain true lifelong learners. The central tenant of social learning theory is the idea that while learning environments influence learners, the learners themselves also help define the learning environment in which they practice [2]. This relationship between learners and skills they acquire via modeling more experienced practitioners is described as situated learning, in which learners participate in and help define communities of practitioners as they move forward to full participation of the sociocultural practice, in this case as physicians [23]. Medical students in their early clinical experiences model more senior student, resident, and attending behavior as it pertains to patient care, communication with patients, families, and other care team members. With observation, they also may replicate how senior practitioners organize their work and instruct junior team members. Role modeling is a fundamental element of clinical medical education [24]. Despite mixed data on learner satisfaction in prior studies, bedside rounds in general seem to have a positive effect on learner behavior and health care delivery [25].

In our study, we aimed to identify and categorize traits, behaviors, and techniques that observer M4 students placed in the learning environment perceived as relevant to creating a favorable teaching rounds experience for all levels of learners. Many highlighted that the leader of rounds’ mood set the tone for the day, emphasizing the benefits of humor and fun. This was illustrated in comments reflecting on joking but also by novelty in approaches to teaching a concept. Chiefs in our study referenced many positive aspects of teaching highlighted in previous studies including providing expectations, promoting goal-setting, engagement, and instilling confidence [3]. Student chiefs also described the importance of both bedside and prepared teaching presentations and promotion of clinical reasoning, all of which have been shown to be evidence-based methods of clinical teaching [4, 6, 12, 13]. Incorporation of evidence-based patient care and teaching was appreciated by our M4s as was teaching to various levels of learners [26, 27]. While debriefing in a feedback setting has been identified as a key component of a clinical co-teaching model [28], our students noted that debriefing after awkward or difficult encounters by their faculty was also invaluable. M4s in our study independently highlighted the importance of the teaching attending recognizing the importance of and opportunity to promote autonomy in patient care activities and communication. This is similar to prior studies showing that doing so can promote the learner team being confident and effective in continuation of care when the attending is physically not present [24].

As students enter clinical learning environments, they begin to absorb knowledge from patients, the health care system, and their medical teaching teams. This experiential learning which takes place in the context of them performing supervised clinical work is vital to their development of progressive independence in clinical reasoning, problem-solving, and communication. The success of this learning environment also heavily relies upon effectiveness as a positive social structure whereby the learners will help contribute to the culture of the team. While a single teaching attending cannot perform all aforementioned techniques during any 1 week, faculty development that supports refining several may well improve the experience a learner has in their teaching environment. Observations made by learners of this environment then are critical in understanding how to create a space in which the most effective learning can take place. These student physicians shortly after joining the team will progress in autonomy and then become teachers themselves, and the attributes and behaviors they choose to model will quickly be modeled by the incoming student physician: it is see one, do one, teach one.
